# miR-708-5p Regulates Myoblast Proliferation and Differentiation

**DOI:** 10.3390/vetsci9110641

**Published:** 2022-11-18

**Authors:** Xueli Xu, Hui Lu, Dong Xu, Zonggang Yu, Nini Ai, Kaiming Wang, Xintong Li, Jun He, Jun Jiang, Haiming Ma, Yuebo Zhang

**Affiliations:** 1 College of Animal Science and Technology, Hunan Agricultural University, Changsha 410128, China; 2 Biological and Environmental Engineering, Yueyang Vocational Technical College, Yueyang 414000, China; 3 Guangdong Laboratory for Lingnan Modern Agriculture, Guangzhou 510000, China

**Keywords:** miR-708-5p, C2C12 cells, proliferation, differentiation, target genes

## Abstract

**Simple Summary:**

Skeletal muscle is a crucial component of an animal’s body. Its growth and development process are modified by a variety of regulatory factors and signaling pathways. It is reported that miR-708-5p is closely related to cancer, osteoporosis, and muscle development. However, the molecular mechanism of miR-708-5p-mediated regulation of myoblast proliferation and differentiation remains unclear. This research aims to investigate the function of miR-708-5p in the proliferation and differentiation of C2C12 myoblasts and analyze its target genes. After interference or overexpression of miR-708-5p, we found that overexpression of miR-708-5p inhibited myoblast proliferation and promoted C2C12 myoblast differentiation. A total of 253 target genes of miR-708-5p were identified. GO and KEGG pathway analysis suggested that these target genes were significantly enriched in terms related to muscle growth and development. *Pik3ca*, *Pik3r3*, and *Irs1* were identified as the key target genes of miR-708-5p. These results deepen our understanding of the molecular mechanism of skeletal muscle development and provide a theoretical foundation for further exploring the mechanism of miR-708-5p-mediated regulation of skeletal muscle development.

**Abstract:**

MicroRNAs (miRNAs) are key regulators involved in the myogenic process in skeletal muscles. miR-708-5p plays an important role in various biochemical and physiological processes, but its function in skeletal myogenesis remain unclear. In this study, we first explored the effects of miR-708-5p on C2C12 proliferation and differentiation by overexpression and interference experiments. Then, we predicted the target genes of miR-708-5p and analyzed their function. We found that miR-708-5p was gradually increased during myoblast differentiation. Overexpression of miR-708-5p significantly inhibited cell proliferation and promoted the differentiation of myoblasts. A total of 253 target genes were predicted using a bioinformatics approach. These genes were significantly enriched in muscle growth-related GO terms and KEGG pathways, such as actin filament organization, actin cytoskeleton organization, PI3K-Akt pathway, insulin pathway, and Jak-STAT pathway. Among them, *Pik3ca*, *Pik3r3*, and *Irs1* were considered to be the key target genes of miR-708-5p. To sum up, miR-708-5p inhibited C2C12 cells proliferation and promoted C2C12 cells differentiation. Its target genes significantly enriched in GO terms and KEGG pathways related to the development and growth of muscle. Our results provided a basis for studies on the function and mechanism of miR-708-5p regulating skeletal muscle growth and development.

## 1. Introduction

Skeletal muscle is the largest tissue in mammals, which represents about 30%–40% of body weight and is formed by muscle fibers and connective tissue [1]. The number and size of muscle fibers relate to the lean meat production, and their types determine the meat quality after slaughter. The growth and development of animal muscle fibers are affected by internal and external conditions, such as genetic, nutritional, and environmental factors [2,3]. Muscle growth is a complicated process, including myoblasts proliferation, migration, differentiation, and fusion [4]. This complex process is precisely regulated by specific myogenic regulatory factors (MRFs), paired box family (Pax), growth factors, cytokine, and other factors [5,6].

MicroRNAs (miRNAs) are small noncoding RNA molecules consisting of about 22 nucleotides and are crucial for the development of animals and plants [7,8]. Generally, the 2-8 nucleotides of miRNA, highly conserved in both plant and mammalian evolution, are referred to as the seed sequence. More and more experiments have shown that miRNAs such as miR-452 [9], miR-206, and miR-1 [10] play a key role in the proliferation and differentiation of myoblasts and affect the growth and development of muscle. In recent years, miR-708-5p has been extensively studied in various diseases, including rheumatoid arthritis, tumors, vascular calcification, and osteoporosis [11,12,13,14]. Shen et al. reported that the expression of miR-708 is decreased in dexamethasone (DEX)-treated C2C12 myoblasts compared with normal differentiated C2C12 myoblasts [15]. The expression of miR-708 was higher in neonatal rat hearts than that in adult rat hearts, and miR-708 facilitates cardiac progenitor cells differentiation in rats [16]. The downregulation of miR-708-5p and the restoration of dystrophin are likely to improve the phenotype of Duchenne and Becker muscular dystrophies [17]. Given that miR-708-5p is possibly involved in the process of muscle development, and the effect of miR-708-5p on muscle cells needs to be deeply explored.

In this experiment, we determined the role of miR-708-5p in C2C12 cells. Furthermore, we analyzed the function of target genes of miR-708-5p using miRWalk online databases to expound the potential mechanisms underlying the role of miR-708-5p in muscle development. Overall, our results indicated that miR-708-5p inhibits the proliferation of C2C12 cells and promotes the differentiation of C2C12 cells. Furthermore, target genes of miR-708-5p were significantly enriched in Gene Oncology (GO) terms and Kyoto Encyclopedia of Genes and Genomes (KEGG) pathways related to the development and growth of muscle, and *Pik3ca*, *Pik3r3*, and *Irs1* were considered to be the key target genes of miR-708-5p.

## 2. Materials and Methods

### 2.1. Cell Culture

C2C12 cell lines were purchased from Anweisci (Shanghai, China) and cultured in complete medium (CM). The CM is composed of 89% Dulbecco’s modified eagle medium (DMEM) (Gibco, Waltham, MA, USA), 10% fetal bovine serum (Gibco, Waltham, MA, USA), and 1% penicillin-streptomycin (Gibco, Waltham, MA, USA). The cell line was cultured in 37 °C incubator containing 5% CO_2_. The cell culture medium was changed to DMEM containing 2% horse serum (Hyclone, Logan, UT, USA) for myogenic differentiation.

### 2.2. Cell Transfection

miR-708-5p mimics (mimics), miR-708-5p mimics control (nc), miR-708-5p inhibitor (inhibitor), and miR-708-5p inhibitor control (inhibitor nc) were purchased from GenePharma (Suzhou, China). The sequences were as follows:
mimics   Forward: 5′-AAGGAGCUUACAAUCUAGCUGGG-3′Reverse: 5′-CAGCUAGAUUGUAAGCUCCUUUU-3′nc   Forward: 5′-UUCUCCGAACGUGUCACGUTT-3′Reverse: 5′-ACGUGACACGUUCGGAGAATT-3′Inhibitor:   5′-CCCAGCUAGAUUGUAAGCUCCUU-3′Inhibitor nc:   5′-CAGUACUUUUGUGUAGUACAA-3′

Additionally, 6-well plates were used to culture C2C12 cells, and C2C12 cells were seeded at a density of 1 × 10^6^ cells per well. Then, the cells were transfected with mimics, nc, inhibitor, and inhibitor nc using Lipofectamine 2000 (Invitrogen, Waltham, MA, USA), as recommended by the manufacturer.

### 2.3. Real-Time Quantitative PCR

Total RNA was separated using RNA simple Total RNA KitI(TIANGEN, Beijing, China), as recommended by the manufacturer. NanoDrop 2000 (Thermo Scientific, Waltham, MA, USA) was used to determine the concentration of RNA. Reverse transcription kit (Thermo Scientific, Waltham, MA, USA) was used for RNA reverse transcription. Real-time quantitative PCR (RT-qPCR) was performed on a CFX connect real-time system (Bio Rad, Hercules, CA, USA) using *U6* and *β-actin* as reference genes. Primer 5.0 was used to design primers according to the sequences downloaded from the NCBI database. All primers were synthesized from Tsingke Biotechnology (Beijing, China). The primers’ sequences are listed in the following table (Table 1). The relative expression of genes was calculated using 2^−△△Ct^.

### 2.4. Cell Proliferation Assays

Cell proliferation was determined through EdU staining and CCK8 assays. Briefly, the C2C12 cells were seeded in 96-well plates when the cells reached 80% confluent and incubated with 50 μM EdU for 2 h. Then, the cells were fixed by 4% paraformaldehyde and permeated with 0.5% tritonX-100. Nuclei were stained by Hoechst 33342 (Beyotime Biotechnology, Shanghai, China). C2C12 cell proliferation was evaluated by Cell Counting kit 8 (Beyotime Biotechnology, Shanghai, China). C2C12 cells were inoculated in 96-well plates and cultured for 0, 24, 48, and 72 h. Then, each well added 10 μL CCK-8 solution at the designated time point. The cells were incubated for 4 h. Subsequently, absorbance at 450 nm was measured using a microplate reader (Multiskan FC, Thermo Scientific, Waltham, MA, USA).

### 2.5. Flow Cytometry

Flow cytometry was used to evaluate cell cycle. We collected the cells transfected with mimics and nc and added 1 mL ice-cold PBS to disperse the cells. The cells were centrifuged to remove PBS, fixed in 1 mL ice-cold 70% ethanol, and stored for 16 h at 4 °C. Then, the cells were collected and centrifuged 2 times at 1000× *g* for 5 min to remove ethanol. The collected cells were treated with 0.5 mL Staining Solution, 10 μL propidium iodide (PI), and 10 μL RNaseA (Yeasen, Shanghai, China) in the dark for 30 min at 37 °C. Finally, the treated cell suspension was measured by the Cytek DxP Athena flow cytometry (Cytek, Fremont, CA, USA).

### 2.6. Immunofluorescence Analysis

Immunofluorescence analysis was carried out to evaluate C2C12 cells differentiation. The differentiated cells were fixed by 4% paraformaldehyde for 30 min, 0.5% triton X-100 for 20 min, and blocked with 5% bovine serum albumin (Bio Froxx, Frankfurt, Germany) for 2 h. Then, the cells were incubated with anti-Myhc monoclonal antibody (1:300, DSHB, Iowa, IA, USA) for 16 h at 4 °C, and then incubated with DyLight 488 goat anti-mouse IgG (1:1000, Abbkine, Wuhan, China) for 2 h. Next, nuclei were stained with DAPI (1:100, Solarbio, Beijing, China) for 10 min. Finally, images were captured on a fluorescence microscope (Axio Vert A1, ZEISS, Oberkochen, Germany). The pictures were analyzed by ImageJ software.

### 2.7. Target Genes Prediction

The target gene of hsa-miR-708-5p and mmu-miR-708-5p was predicted by miRWalk “http://mirwalk.umm.uni-heidelberg.de (accessed on 29 August 2022)” online software. The miRWalk online software integrates the predicted targets from miRDB, TargetScan, and miRTarbase. We used Venny 2.1.0 “https://bioinfogp.cnb.csic.es/tools/venny/index.html (accessed on 29 August 2022)” online software to intersect the predicted target genes. To identify functional categories of the candidate target genes of miR-708-5p, GO enrichment analysis and KEGG pathway enrichment analysis were performed using KOBAS “http://kobas.cbi.pku.edu.cn (accessed on 14 September 2022)” online software. The Q value is the corrected *p*-value. Q value < 0.05 and more than three gene counts were examined. The ratio of the number of target genes enriched in this pathway to the number of all target genes annotated in the same pathway is the enrichment factor. Bioinformatics website “http://www.bioinformatics.com.cn (accessed on 16 September 2022)” was used to draw pictures.

### 2.8. The PPI and miRNA–mRNA Network Construction

STRING online software (version 11.5) “https://cn.string-db.org (accessed on 20 October 2022)” was used to determine the protein–protein interaction (PPI) network. Cytoscape was used to visualize the results of STRING. Nodes above 10 were included in the screen scope.

### 2.9. Statistical Analyses

ImageJ software was used for cell counting. Statistical analysis of the data was performed with one-way ANOVA or Student’s *t*-test using IBM SPSS 22.0 software. The data were indicated as mean ± standard error. Statistically significant differences were considered at *p* < 0.05. * *p* < 0.05 means significant difference and ** *p* < 0.01 means very significant difference.

## 3. Results

### 3.1. miR-708-5p Inhibits C2C12 Cells Proliferation

**Figure 1:**
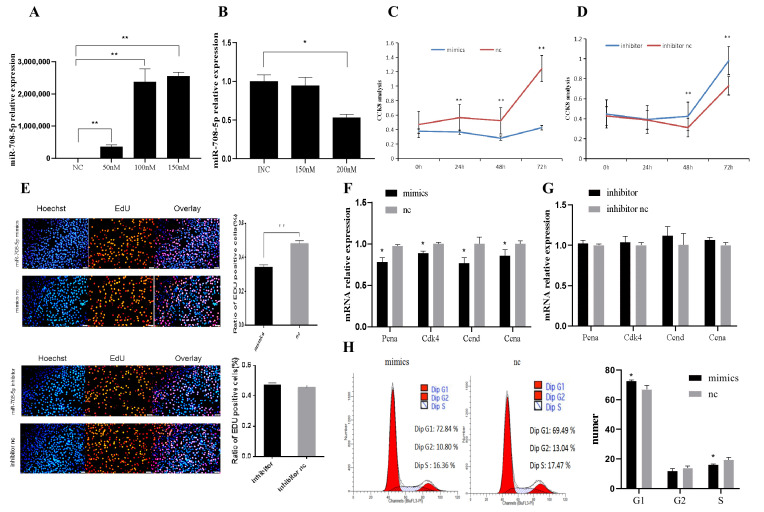
miR-708-5p inhibits C2C12 cell proliferation. (**A**,**B**) Relative expression levels of miR-708-5p were detected by RT-qPCR 24 h after transfection with miR-708-5p mimics (mimics), miR-708-5p mimics control (nc), miR-708-5p inhibitor (inhibitor), and inhibitor control (inhibitor nc). (**C**,**D**) Cell viability was measured using CCK8 after transfection with mimics, nc, inhibitor, and inhibitor nc. (**E**) EdU assay was carried out to detect the effect of miR-708-5p inhibition and overexpression on proliferation of C2C12 cells. (**F**,**G**) Expression levels of the genes related to cell proliferation were detected by RT-qPCR. (**H**) The cell cycle distribution of C2C12 cells was determined by flow cytometry after transfection with mimics or nc. * *p* < 0.05 and ** *p* < 0.01. Results are presented as mean ± S.E.M. n = 3.

As shown in Figure 1A,B, we found that transfection of 50 nM, 100 nM, and 150 nM mimics all had the overexpression efficiency, and transfection of 200 nM inhibitor had the highest inhibition efficiency (*p* < 0.05). Therefore, transfection of mimics or inhibitor was performed at 100 nM or 200 nM in subsequent experiments, respectively. The CCK8 analysis results indicated that miR-708-5p inhibited C2C12 cells proliferation (Figure 1C,D). The EdU results indicated that the rate of EdU-positive cells decreased significantly after overexpression of miR-708-5p (*p* < 0.01), while inhibition of miR-708-5p was not significantly different (Figure 1E). As shown by the result of RT-qPCR, the expression level of *Cdk4*, *Pcna*, *Ccnd*, and *Ccna* in the mimics group was significantly lower than that in miR-708-5p mimics control (nc) (*p* < 0.05) (Figure 1F), while these genes’expression levels in the inhibitor group were not significantly different from those in the miR-708-5p inhibitor control (inhibitor nc) (*p* > 0.05) (Figure 1G). To further confirm the effect of miR-708-5p in cell proliferation, cell cycle was detected using flow cytometry. The results showed that the number of G1 phase cells in the mimics group was much higher than that in nc (*p* < 0.05), and the number of S phase cells was significantly lower than that in nc (*p* < 0.05) (Figure 1H). In general, the above results suggested that miR-708-5p could inhibit C2C12 cell proliferation.

### 3.2. miR-708-5p Promotes C2C12 Cells Differentiation

**Figure 2:**
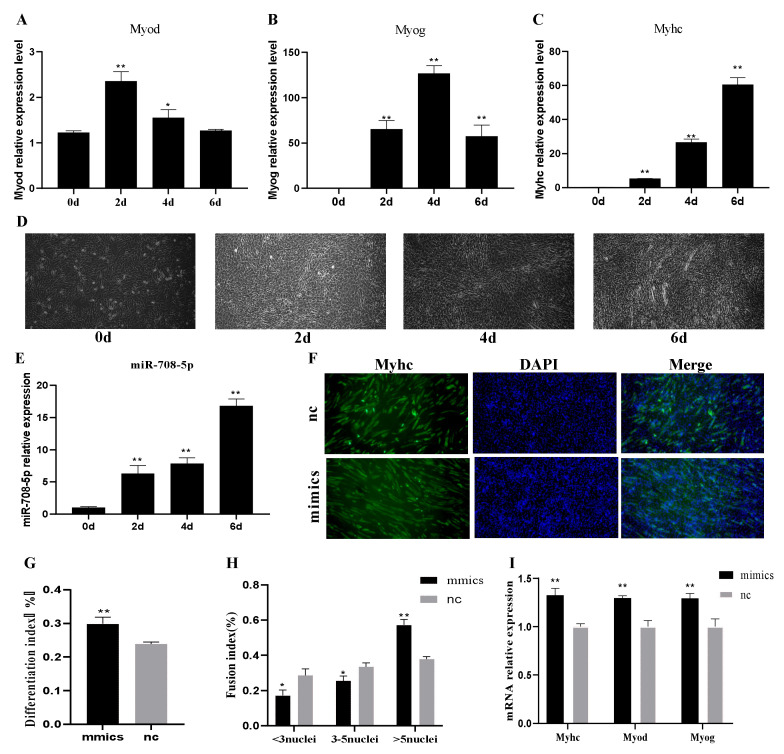
miR-708-5p promotes C2C12 cell differentiation. (**A**–**C**) Relative expression levels of *Myod*, *Myog*, and *Myhc* in C2C12 myoblasts after differentiation for 0 (0 d), 2 (2 d), 4 (4 d), and 6 (6 d) days. (**D**) Morphological change of C2C12 myoblasts after differentiation for 0 d, 2 d, 4 d, and 6 d. (**E**) Relative expression levels of miR-708-5p in C2C12 myoblasts after differentiation in four time point. (**F**) Immunofluorescence analysis of Myhc in C2C12 myoblasts at 4th day of differentiation. (**G**,**H**) The differentiation index and fusion index of myoblasts after transfection with mimics and nc. (**I**) Relative expression levels of *Myod*, *Myog*, and *Myhc* were detected by RT-qPCR at 6th days of myoblasts differentiation after transfection with mimics and nc. * *p* < 0.05 and ** *p* < 0.01. Results are presented as mean ± S.E.M. n = 3.

As shown in Figure 2A,B, *Myod* and *Myog* were most-strongly expressed at 2 d and 4 d, respectively, and then both gradually downregulated. *Myhc*, a late differentiation marker gene, was increasingly expressed with differentiation progress (Figure 2C). In addition, we used a microscope to capture the pictures of differentiated cells. As shown in Figure 2D, the cells were more than 80% confluent at 0 d, and a few cells fused at 2 d. A large number of myotubes appeared on the 4th day of differentiation, and the myotubes became longer and thicker at 6 d. These data indicated that C2C12 cells were well differentiated. To explore the effect of miR-708-5p on C2C12 cell differentiation, we detected the endogenous miR-708-5p in differentiated C2C12 myoblasts. It showed that the miR-708-5p expression level was upregulated during myogenic differentiation in C2C12 myoblasts (Figure 2E). The results of immunofluorescence analysis of *Myhc* suggested that the myotubes formation and differentiation index was significantly increased in the mimics group compared to nc (*p* < 0.01) (Figure 2F–H). As shown by the results of RT-qPCR, the expressions of *Myhc*, *Myod*, and *Myog* at 6 days of myoblasts differentiation in the mimics group were significantly higher than that in the nc group (*p* < 0.01) (Figure 2I). These results indicated that miR-708-5p could promote C2C12 cells differentiation.

### 3.3. Target Gene Prediction of miR-708-5p

**Figure 3:**
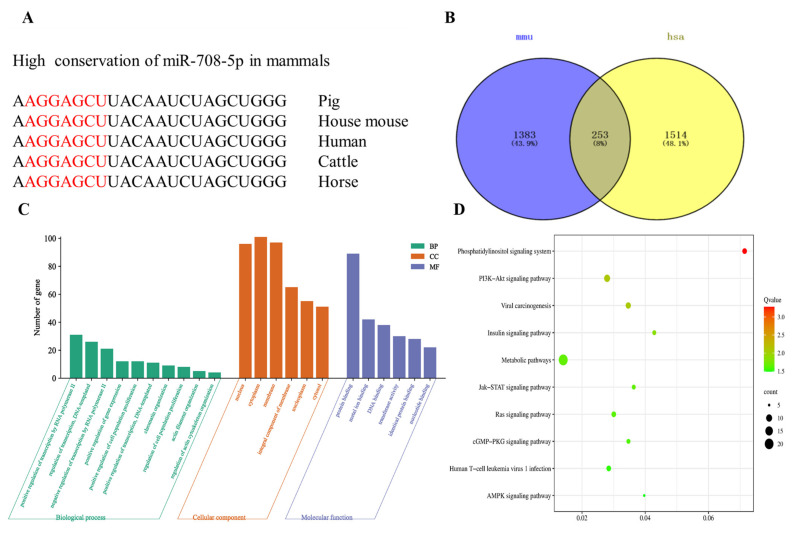
Enrichment analysis of target gene of miR-708-5p. (**A**) Conservation analysis of miR-708-5p mature sequence among five species. (**B**) A Venn diagram showing the overlap between the target genes of mmu-miR-708-5p and hsa-miR-708-5p predicted by miRWalk. (**C**) GO enrichment analysis of the candidate target genes of miR-708-5p. (**D**) KEGG enrichment analysis of the candidate target genes of miR-708-5p.

miR-708-5p was highly conserved among human (*Homo sapiens*), house mouse (*Mus musculus*), pig (*Sus scrofa*), cattle (*Bos taurus*), and horse (*Equus caballus*) based on nucleotide sequences (Figure 3A). We used miRWalk to predict the target genes of hsa-miR-708-5p and mmu-miR-708-5p. In total, 253 target genes were overlapped (Supplementary Material, Table S1). The Venn diagram is shown in Figure 3B. The 253 target genes were enriched in 68 GO terms, including 22 biological processes, 26 cellular components, and 20 molecular function terms. As shown in Figure 3C, we listed 20 typical enriched GO terms (Q value < 0.05). In the biological process, the target genes of miR-708-5p are mainly involved in the regulation of transcription by RNA polymerase II, actin filament organization, regulation of actin cytoskeleton organization, the regulation of transcription, DNA-templated and positive regulation of gene expression, and cell population proliferation. In the cellular component, these genes concentrated distribution on the nucleus, cytoplasm, and membrane. The molecular function was concentrated in protein binding, DNA binding, metal ion binding, and transferase activity. The GO enrichment results showed that the target genes mainly played a role in binding, regulation of actin cytoskeleton organization, actin filament organization, and pretranscriptional regulation. To identify signaling pathways in which the 253 candidate target genes were enriched, we performed KEGG enrichment analysis. A total of 34 pathways were annotated (Q value < 0.05), and the top 10 typically enriched KEGG pathway terms are shown in Figure 3D. The target genes were significantly enriched in several signaling pathways, such as metabolic pathways, PI3K-Akt signaling pathway, cGMP-PKG signaling pathway, insulin signaling pathway, and AMPK signaling pathway, which suggests that those genes may perform their function through these pathways.

### 3.4. The PPI and miRNA–mRNA Network Construction

**Figure 4:**
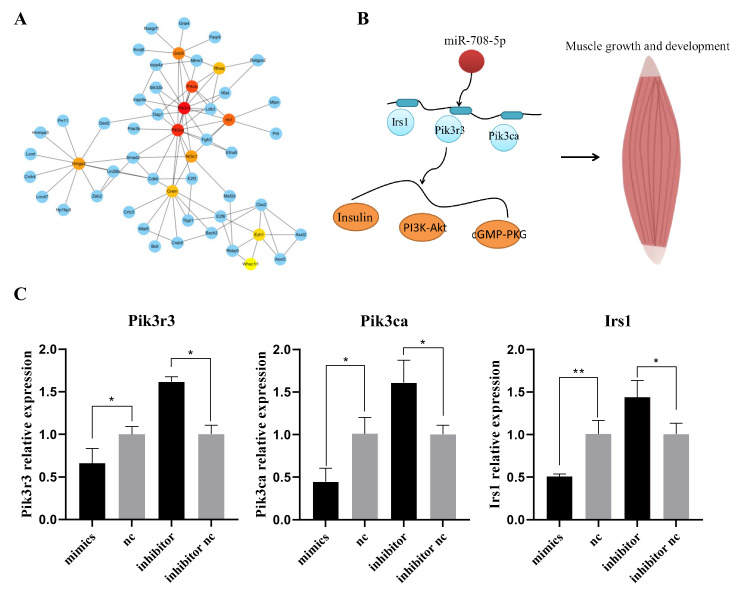
Construction of protein–protein interaction (PPI) network and miRNA–mRNA network. (**A**) The PPI network of the target genes of miR-708-5p. (**B**) The miRNA–mRNA network between miR-708-5p and the key target genes. (**C**) The expression level of *Pik3r3*, *Pik3ca*, and *Irs1* were detected by RT-qPCR 24 h after transfection with mimics, nc, inhibitor, and inhibitor nc. * *p* < 0.05 and ** *p* < 0.01. Results are presented as mean ± S.E.M. n = 3.

In total, 253 target genes were analyzed in STRING online software. The PPI network was constructed by Cytoscape, and the top 10 hub-genes were *Pik3r3*, *Pik3ca*, *Prkce*, *Irs1*, *Gnb5*, *Nr3c1*, *Hmga2*, *Crem*, *Rhoq*, and *Ezh1* (Figure 4A). According to the literature, we selected three genes associated with myogenic differentiation. Among these genes, *Pik3r3*, *Pik3ca*, and *Irs1* were selected to draw the miRNA–mRNA network (Figure 4B). As the results of qRT-PCR (Figure 4C), the expression level of *Pik3r3*, *Pik3ca*, and *Irs1* was significantly decreased after overexpression of miR-708-5p (*p* < 0.05), and the inhibition group showed an opposite result (*p* < 0.05). This result indicated that *Pik3r3*, *Pik3ca*, and *Irs1* might be the key target genes of miR-708-5p.

## 4. Discussion

In recent years, miR-708 has been widely studied in various human cancers. Its expression in tumor cells is closely related to cell proliferation, migration, invasiveness, and chemosensitivity, which make miR-708 receive more attention. According to the literature, miR-708-5p can significantly inhibit the proliferation of tumor cells [18,19,20]. Sui et al. reported that miR-708-5p inhibits the growth and invasion of osteosarcoma cells [21]. MCM3AP-AS1 regulates the proliferation and apoptosis of gastric cancer cells by targeting miR-708-5p [22]. A large number of studies has shown that miRNAs play a very important role in the growth and development of skeletal muscle [23,24,25]. In this study, we investigated the role of miR-708-5p in skeletal muscle development. Given that the C2C12 cell line is a suitable cell model for researching muscle differentiation in vitro [26], we studied the function of miR-708-5p in C2C12 cells.

The results of CCK8 analysis (Figure 1C,D), EdU staining (Figure 1E), and the expression levels of *Pcna*, *Cdk4*, *Ccnd*, and *Ccna* (Figure 1F,G) indicated that miR-708-5p inhibited cell proliferation. The expression of *Pcna* directly reflects the proliferation of cells. *Cdk4*, *Ccnd*, and *Ccna* are key factors in the cell proliferation process [27,28]. Although the interference efficiency is 45%, the interference effect was not good. This may be caused by the originally low expression level of miR-708-5p in C2C12 cells. Thus, in the next experiment, the interference experiment was canceled. In this study, we found that most of the cells in the mimics group were blocked in the G1 phase, but the rate of cells in the G2 phase significantly decreased (Figure 1H). We speculate that overexpression of miR-708-5p blocks the progress of cell cycle and inhibits the replication of DNA in the S phase, thus inhibiting cell proliferation.

*Myod*, *Myog*, and *Myhc* are differentiation marker genes. Their expression is an index to detect the degree of muscle differentiation. Our results indicated that C2C12 cells were well-differentiated (Figure 2A–D) [29,30,31]. Given that miR-708-5p was increased during myogenic differentiation in C2C12 cells (Figure 2E), we further explored the effects of miR-708-5p on myogenesis. Here, transfection of mimics significantly increased the differentiation index (Figure 2G), fusion index (Figure 2H), and the expression levels of *Myod*, *Myog*, and *Myhc* (Figure 2I), which suggested that miR-708-5p could dramatically promote myogenic differentiation. As we all know, cell arrest in the G1 phase is a key step in the process of differentiation. Li et al. found that a high expression level of porcine miR-95 in C2C12 cells could arrest cells in the G1 phase, thus promoting the cells to enter the differentiation phase [32]. Our results also showed that a high expression level of miR-708-5p could arrest cells in the G1 phase and promote C2C12 cells constrained into the differentiation stage. These results indicated that miR-708-5p might be a positive regulator of skeletal muscle differentiation.

miR-708-5p was highly conserved among mouse, human, pig, cattle, and horse, with a homology of 100% (Figure 3A), suggesting that miR-708-5p has similar biological function in different species. To explore the function and pathway of miR-708-5p in muscle, miRWalk was used to predict the target genes of miR-708-5p, and GO and KEGG pathway enrichment analysis were used to analyze these target genes. The GO enrichment results showed that the target genes of miR-708-5p were significantly enriched in actin filament organization and regulation of actin cytoskeleton organization and other biological processes related to the growth and development of muscle (Figure 3C). The candidate target genes were significantly enriched in the PI3K-Akt signaling pathway, insulin signaling pathway, and cGMP-PKG signaling pathway (Figure 3D), such as *Cdk6*, *Fgfr2*, *Mef2d*, *Pik3ca*, *Irs1*, and *Pik3r3*. *Cdk6* participates in cell cycle regulation; D-type cyclins together with *Cdk6* and *Cdk4* drive cell proliferation [33]. In skeletal muscle stem cells, *Fgfr2* plays an important role in the myogenesis [34]. *Mef2d* is a crucial transcriptional activator of myogenin expression [35]. In this study, *Pik3ca, Pik3r3*, and *Irs1* were considered to be the key target genes of miR-708-5p (Figure 4C). Among these genes, *Pik3ca* is related to muscular hemihyperplasia [36]. As the main regulator of the PI3K-Akt signaling pathway, *Pik3r3* plays a regulatory role in differentiation and apoptosis in cardiomyocytes [37]. *IRS1* plays an important role in skeletal muscle, and it is essential for the normal growth and differentiation of muscle fibers [38].

## 5. Conclusions

In the present study, we found that miR-708-5p suppressed C2C12 cells proliferation and promoted their myogenic differentiation. The target genes of miR-708-5p were significantly enriched in muscle development-related GO terms and KEGG pathways. *Pik3ca*, *Pik3r3*, and *Irs1* were considered to be the key target genes of miR-708-5p. We preliminarily revealed the function of miR-708-5p in the process of C2C12 cell proliferation and differentiation and laid a foundation for further study of miR-708-5p on the regulatory mechanism of muscle growth and development.

## Figures and Tables

**Table 1:** Primers for RT-qPCR.

Gene	Primers	Primer Sequence (5′→3′)	Annealing Temperature (°C)
*U6*	F	CTCGCTTCGGCAGCACA	60
	R	AACGCTTCACGAATTTGCGT	
*β-actin*	F	CTTGCGTTTGGCTATCCGTG	60
	R	TCTCCGAAGCCAGCAATTCA	
miR-708-5p	F	TCGGCAGGAAGGAGCTTACAAT	60
	R	CTCAACTGGTGTCGTGGA	
miR-708-5p	RT	CTCAACTGGTGTCGTGGAGTCGGCAATTCAGTTGAGCCCAGCTA	
*Cdk4*	F	CGAGCGTAAGGCTGATGGAT	60
	R	CCAGGCCGCTTAGAAACTGA	
*Ccna*	F	GAGCTCCCAAGCTCTACTGC	60
	R	TTTTCATGGGCAGTCCTGGT	
*Ccnd*	F	GAAGGAGGTAAGGGAAGCACTC	60
	R	TAGCGCTCCTCGATGGTCAA	
*Pcna*	F	GCCGAGACCTTAGCCACATT	60
	R	GTAGGAGACAGTGGAGTGGC	
*Myhc*	F	AGCCAAGTGCTAGACCGAAA	60
	R	CGTCGTGAGCCCAAAACTTC	
*Myog*	F	CAGCCCAGCGAGGGAATTTA	60
	R	GGTCAGGGCACTCATGTCTC	
*Myod*	F	AAGACGACTCTCACGGCTTG	60
	R	GCAGGTCTGGTGAGTCGAAA	
*Pik3r3*	F	TTGCAGACGGGGAAGTGAAG	60
	R	GACGTTGAGGGAGTCGTTGT	
*Pik3ca*	F	CTCAGCTCTCACCCTCCTCT	60
	R	GGTCTCTCTTTCCGCTCACA	
*Irs1*	F	AATGAGGGCAACTCCCCAAG	60
	R	ATTGACGATCCTCTGGCTGC	

## Data Availability

The data set analyzed for the current study is available from the corresponding author upon reasonable request.

## Supplementary Materials

The following supporting information can be downloaded at: www.mdpi.com/vetsci-09-00641/s1, Table S1: overlapped target genes of hsa-miR-708-5p and mmu-miR-708-5p.
